# A double deletion prevents replication of the pestivirus bovine viral diarrhea virus in the placenta of pregnant heifers

**DOI:** 10.1371/journal.ppat.1010107

**Published:** 2021-12-08

**Authors:** Jolene Carlson, Robert Kammerer, Jens Peter Teifke, Julia Sehl-Ewert, Christiane Pfarrer, Gregor Meyers

**Affiliations:** 1 Institut für Immunologie, Friedrich-Loeffler-Institut, Greifswald-Insel Riems; 2 Abteilung für experimentelle Tierhaltung und Biosicherheit, Friedrich-Loeffler-Institut, Greifswald-Insel Riems; 3 Institute for Anatomy, University of Veterinary Medicine, Hannover, Germany, University of Veterinary Medicine Hannover, Hannover, Germany; USDA-ARS National Animal Disease Center, UNITED STATES

## Abstract

In contrast to wild type bovine viral diarhea virus (BVDV) specific double deletion mutants are not able to establish persistent infection upon infection of a pregnant heifer. Our data shows that this finding results from a defect in transfer of the virus from the mother animal to the fetus. Pregnant heifers were inoculated with such a double deletion mutant or the parental wild type virus and slaughtered pairwise on days 6, 9, 10 and 13 post infection. Viral RNA was detected via qRT-PCR and RNAscope analyses in maternal tissues for both viruses from day 6 p.i. on. However, the double deletion mutant was not detected in placenta and was only found in samples from animals infected with the wild type virus. Similarly, high levels of wild type viral RNA were present in fetal tissues whereas the genome of the double deletion mutant was not detected supporting the hypothesis of a specific inhibition of mutant virus replication in the placenta. We compared the induction of gene expression upon infection of placenta derived cell lines with wild type and mutant virus via gene array analysis. Genes important for the innate immune response were strongly upregulated by the mutant virus compared to the wild type in caruncle epithelial cells that establish the cell layer on the maternal side at the maternal–fetal interface in the placenta. Also, trophoblasts which can be found on the fetal side of the interface showed significant induction of gene expression upon infection with the mutant virus although with lower complexity. Growth curves recorded in both cell lines revealed a general reduction of virus replication in caruncular epithelial cells compared to the trophoblasts. Compared to the wild type virus this effect was dramtic for the mutant virus that reached only a TCID_50_ of 1.0 at 72 hours post infection.

## Author summary

Here we report on animal studies elucidating mechanisms preventing the transfer of a double deletion mutant of a pestivirus to the fetus in pregnant heifers. This mutant lacks both known factors engaged in blocking the innate immune response to pestiviral infection. As shown also in earlier studies, this mutant was not detected in the fetuses at any of the tested time points in contrast to the wild-type (wt) virus. However, similar to the wt the mutant was detected in a large variety of different maternal tissues. The only exception was the placenta where only wt but not mutant virus was detected. Using gene array analyses we showed that infection of two cell lines derived either from the maternal or the fetal site of the maternal-fetal interface with the mutant virus induces a significant antiviral gene expression response. The reaction of cells from the maternal side was more complex and virus replication in these cells was reduced, almost completly blocking the mutant virus. These results support the hypothesis that replication of the mutant virus is blocked in the placenta due to a highly active innate immune response and the prevention of replication also blocks transfer of the virus to the fetus.

## Introduction

Pestiviruses are responsible for diseases of animals that are of great economic importance [1–6]. Classical swine fever virus (CSFV), two types of bovine viral diarrhea virus (BVDV-1 and BVDV-2), and border disease virus of sheep (BDV) and a variety of isolates from other animal species belong to this group of viruses, and are classified as one genus within the virus family *Flaviviridae* [7]. As for all members of the family, the pestivirus genome consists of a positive sense single stranded RNA molecule that contains one long open reading frame (ORF) coding for all known viral proteins [5,8]. Protein expression occurs via translation of the genomic RNA into a polyprotein that is co- and post-translationally processed by viral and cellular proteases into the mature virus proteins. Within the polyprotein the individual gene products are arranged in the order NH2- N^pro^/C/E^rns^/E1/E2/p7/NS2/NS3/NS4A/NS4B/NS5A/NS5B-COOH [5,8]. Protein C, and the glycoproteins E^rns^, E1 and E2 represent structural components of the enveloped pestivirus virion [9]. E2 and to a lesser extent, E^rns^ were found to be targets for antibody neutralization [10–14].

E^rns^ lacks a typical transmembrane sequence or another kind of a membrane anchor known for envelope proteins and is secreted in considerable amounts from infected cells [15]. Our analyses showed that the C-terminal part of the protein functions as a novel type of membrane anchor consisting of a long amphipathic helix binding in plane to the surface of the lipid bilayer [16–19]. E^rns^ was reported to interact with carbohydrate structures on the surface of target cells [20–23]. As a highly interesting feature, E^rns^ exhibits RNase activity [24–28]. It has been shown that the E^rns^ RNase is able to block the cellular interferon response to extracellular double stranded RNA [29–33]. In all these reports, the RNase activity of E^rns^ was shown to be essential for the observed effects. Different results were communicated by one group for CSFV. These authors claimed that the RNase activity was dispensable for blocking of the dsRNA-induced interferon response, whereas glycosylation of the protein was described to be crucial [34–36].

A second highly interesting polypeptide with enzymatic activity is N^pro^, the first protein encoded by the long ORF. N^pro^ is an unusual cysteine protease [37,38] that cleaves at its own carboxyterminus and thus generates the aminoterminal end of the capsid protein. There is no further substrate known for this protease. 3D structure analyses have shown that the carboxyterminus of the protein stays bound to the active side after cleavage thereby blocking access to any potential further substrate so that it is obvious that the release N^pro^ protein is no longer a functional protease. The N^pro^ coding region can be deleted from the viral genome without dramatic influence on the growth characteristics of the virus [39] but results in an attenuated virus [40,41]. It was first reported for CSFV that the deletion of the N^pro^ coding part of the genome results in a mutant that induces an interferon response of the infected cell; therefore, N^pro^ was proposed to interfere with the innate immune response of the cell [42,43]. Loss of repression of interferon induction was also reported for a BVDV N^pro^ deletion mutant and cytopathic BVDV that express normal amounts of functional N^pro^ [44–46]. Similarly, a cytopathic CSFV generated by site directed mutagenesis was no longer able to repress interferon induction as determined by the expression of the Mx protein in infected PK-15 cells [44–47]. The function of N^pro^ with regard to interference with IFN-I secretion was independent of its proteolytic activity [44]. The observed repression of an interferon response by noncytopathic BVDV was correlated with the absence of interferon regulatory factor 3 (IRF3) based activation of gene expression [48]. It was published later on that N^pro^ is able to induce degradation of IRF3 via the proteasome [45,49–51].

Isolates of BVDV, classified now as pestivirus species A (old BVDV-1) and B (old BVDV-2) [52], can either be non-cytopathic (non-cp) or cytopathic (cp), according to their phenotype in tissue culture. Only the non-cp biotype is able to establish lifelong persistent infections upon infection of pregnant heifers and diaplacental transfer of the virus to the fetus [5]. We have shown before that double deletion mutants of BVDV (ddBVDV) with most of the N^pro^ coding sequence eliminated and an E^rns^ gene deficient of one codon crucial for its RNase activity were not able to establish persistent infection in the fetus upon infection of pregnant heifers [53]. This result was not due to blocked replication of the mutants in the fetus since circumvention of the maternal system accomplished by introducing the viruses directly in the amniotic fluid resulted in replication of the mutants and a very prominent interferon response (type 1 or, according to later results, type3 [54]) in the fetuses and death of the fetuses. Moreover, dissemination of the ddBVDV in calves was shown to be comparable to wild type BVD. Taken together, these data argue in favor of a barrier that prevents virus transmission to the fetus in the pregnant animals. We therefore conducted studies with pregnant heifers to compare the dissemination of wt and ddBVDV at very early time points post infection. We show that the genomes of both viruses are found in various tissues of the dams. No significant differences with regard to the number of virus positive tissues or the detected amount of genomic RNA to a given time point p.i. was detected between the two viruses. In contrast, only RNA from wt BVDV was detected in the fetal tissues. Importantly, also samples derived from the placenta were positive only in wt BVDV infected animals whereas ddBVDV RNA was not detected.

## Results

### Animal study

**Fig 1:**
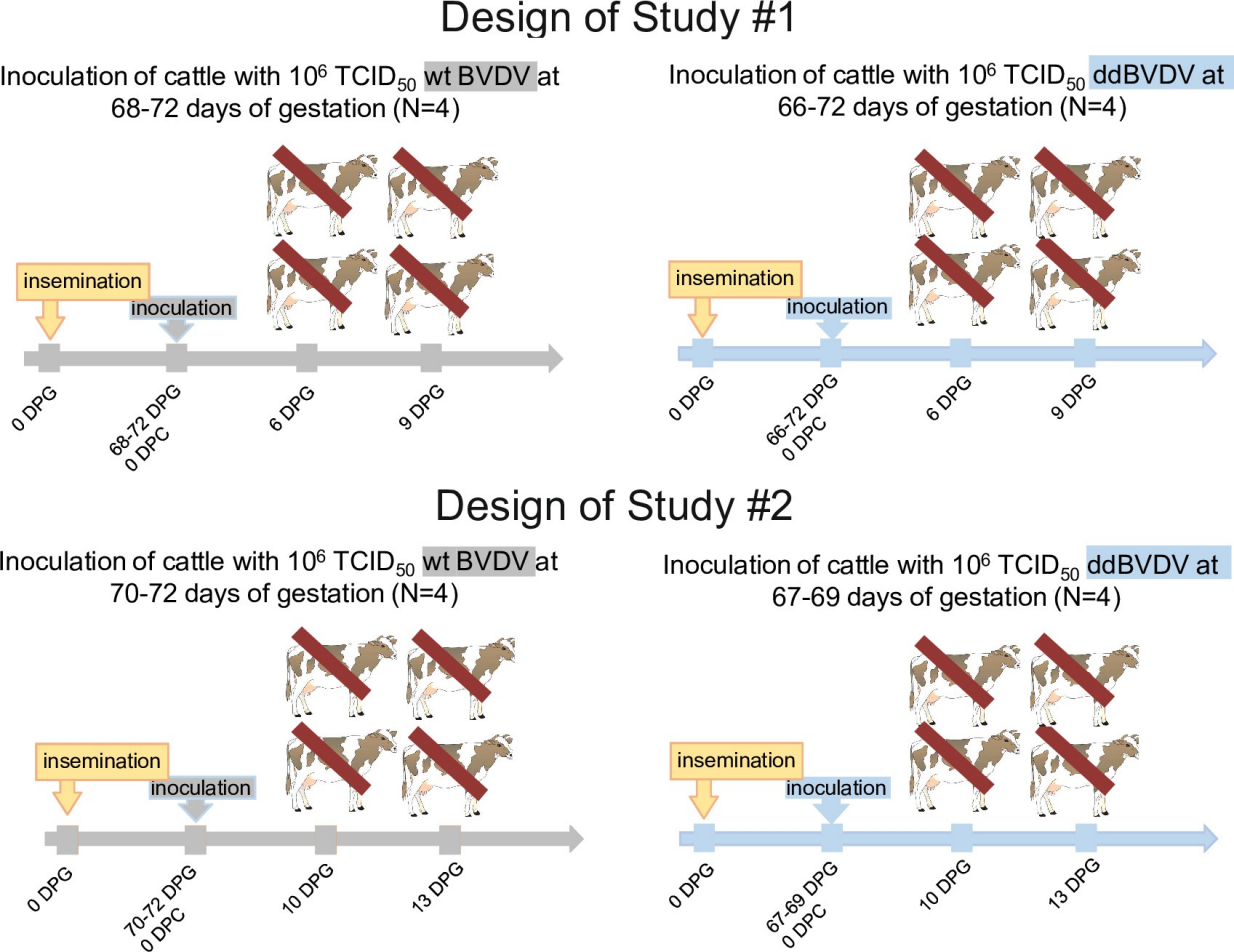
Study design configuration for wt BVDV and ddBVDV pregnant bovine trial. DPG: days post gestation: DPC: days post challenge infection.

The noncytopathic BVDV Ib isolate KE9 (wt BVDV) was attenuated by two deletions that eliminate viral antagonists of the innate immune response [55]. The genome of the resulting mutant ddBVDV exhibits deletions of the complete N^pro^ coding region except the first four codons and codon 349, encoding an active side histidine in the RNase domain of E^rns^. These two deletions eliminate two factors that normally interfere with the innate immune response of infected cells [29–33,42–46,56] and prevent the transmission in pregnant heifers of the ddBVDV to the fetus [53]. In a previous study, calves were infected with ddBVDV and dissemination and tissue tropism of this virus were analyzed by immunohistochemistry at 4 and 6 days p.i. The ddBVDV mutant showed a similar dissemination pattern and tissue distribution as observed before in experimental infection with low virulence BVDV strains (Elisabeth Liebler-Tenorio FLI Jena, personal communication and [57]). Thus, the available data strongly indicated the presence of a barrier in the mother animals, at which ddBVDV is blocked from transfering to the fetus. To analyze the influence of the deletions in detail, we compared distribution of wt and ddBVDV in pregnant heifers early after intramuscular infection. In total, 16 pregnant heifers around 66–72 days of gestation were inoculated in two consecutive studies (8 with ddBVDV and 8 with wt BVDV) (Fig 1 and Table 1). Each animal received 10^6^ TCID_50_ of virus via the intramuscular route and was monitored thereafter daily for clinical signs according to a clinical score matrix (evaluation of general health, behaviour, breathing, cough and body temperature with scores of 0 to 10 for each parameter). No systemic or local adverse reactions related to the ddBVDV mutant or the corresponding wt virus occurred. Fever (40.2°C) was only present in one heifer (#685) at 8 days post-infection with the wt virus. Heifer 685 had a slightly reduced appetite and watery eyes. She was attentive but less active. Her respiration rate was slightly above the baseline of 30 respirations per minute. No cough was present. Her total clinical score was still very low (score = 4). No severe or significant signs of disease or indication for abortion were observed. Nasal swabs were analyzed for viral RNA using a standard diagnostic real time RT-PCR [58]. Only one animal infected with ddBVDV showed virus shedding (957, day 6 p.i.), whereas the wt virus was found in nasal discharge from all wt virus-infected animals of the second study on day 6 and 3 heifers out of 4 on day 10. Blood samples were taken on day 6 and the day of slaughter in study #1 and on days 6, 10 and day of slaughter in study #2.

**Table 1:** Summary of heifer gestation and infection dates.

Bovine ID	Infected on day of gest.	Slaughter	Day of gestation at slaughter	
**Study #1**				
**708**	68	9 dpi	77	**ddBVDV**
**747**	66	9 dpi	75	
**751**	71	6 dpi	77	
**752**	71	6 dpi	77	
**685**	68	9 dpi	77	**wt BVDV**
**719**	70	9 dpi	79	
**741**	71	6 dpi	77	
**745**	72	6 dpi	78	
**Study #2**				
**957**	69	10 dpi	79	**ddBVDV**
**983**	67	13 dpi	80	
**990**	68	13 dpi	81	
**999**	69	10 dpi	79	
**000**	71	13 dpi	84	**wt BVDV**
**126**	70	10 dpi	80	
**519**	70	10 dpi	80	
**947**	72	13 dpi	85	

All wt virus infected animals tested positive for viral genome on day 6 p.i. and 3 out of 4 on day 9 p.i. (study #1) and days 6, 10, 13 (study#2). In contrast, the blood of ddBVDV infected heifers stayed negative in study #1 and only 4 samples were positive in study#2 (animal 983 on days 6 and 13, animals 957 and 990 on day 10).

**Fig 2:**
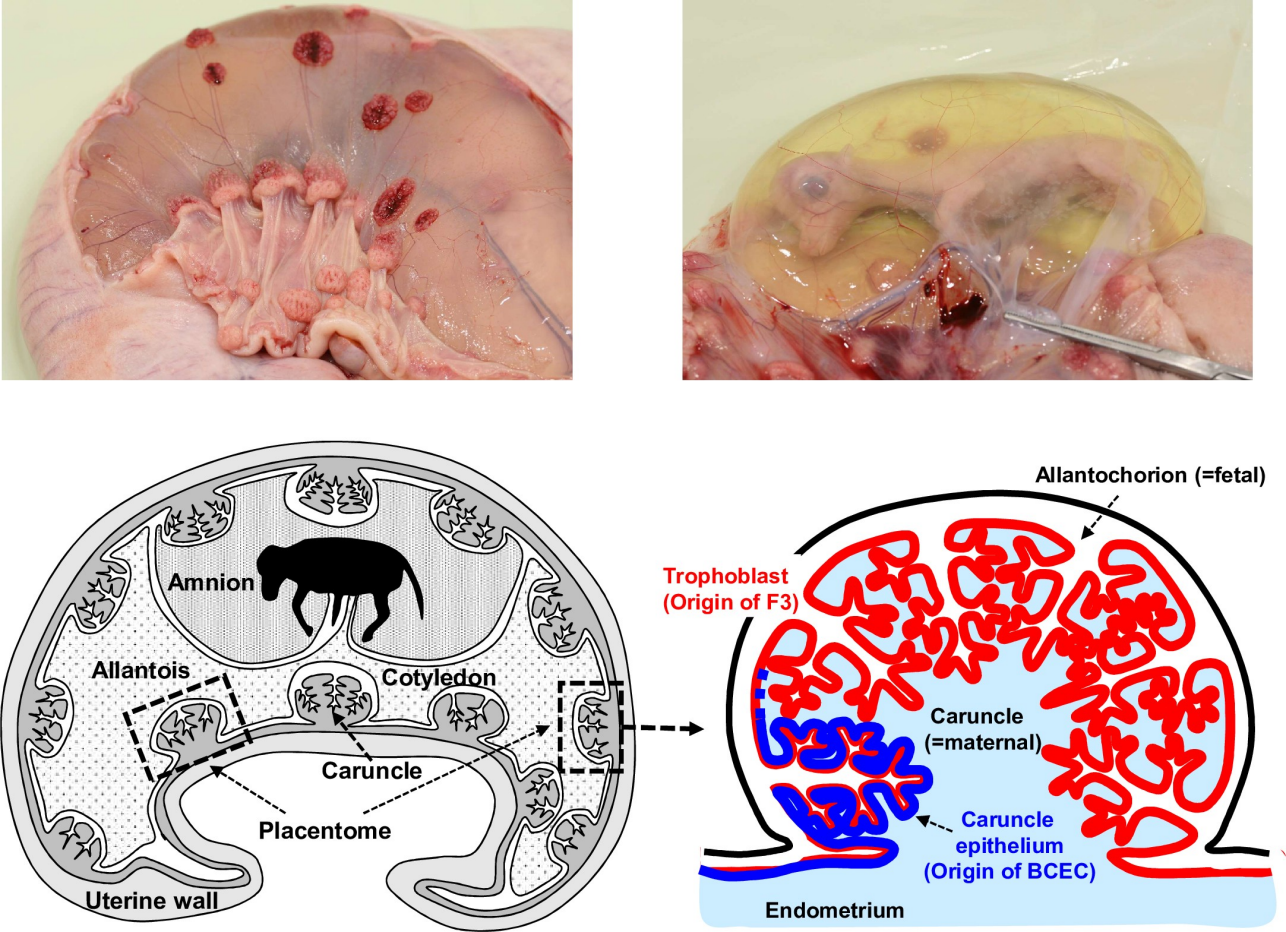
Extracted bovine uteruses and schematic anatomy of bovine placenta. The photographs in the upper part show uteruses extracted from the heifers during necropsy. The dark red structures visible on the surface of the organs shown on the upper left photograph represent the cotyledons as indicated by an arrow. Another arrow points at a caruncle. The anatomy of the bovine placenta is illustrated in the schemes below. The drawing on the left shows the uterus with the fetus whereas the scheme on the right represents a magnified image of the placentome (marked by a box in the left scheme) illustrating its location and how the structures are in contact represented by the fetal (red) and maternal (blue) epithelium, from which the cell lines used in our analyses were derived (F3 and BCEC, respectively).

**Fig 3:**
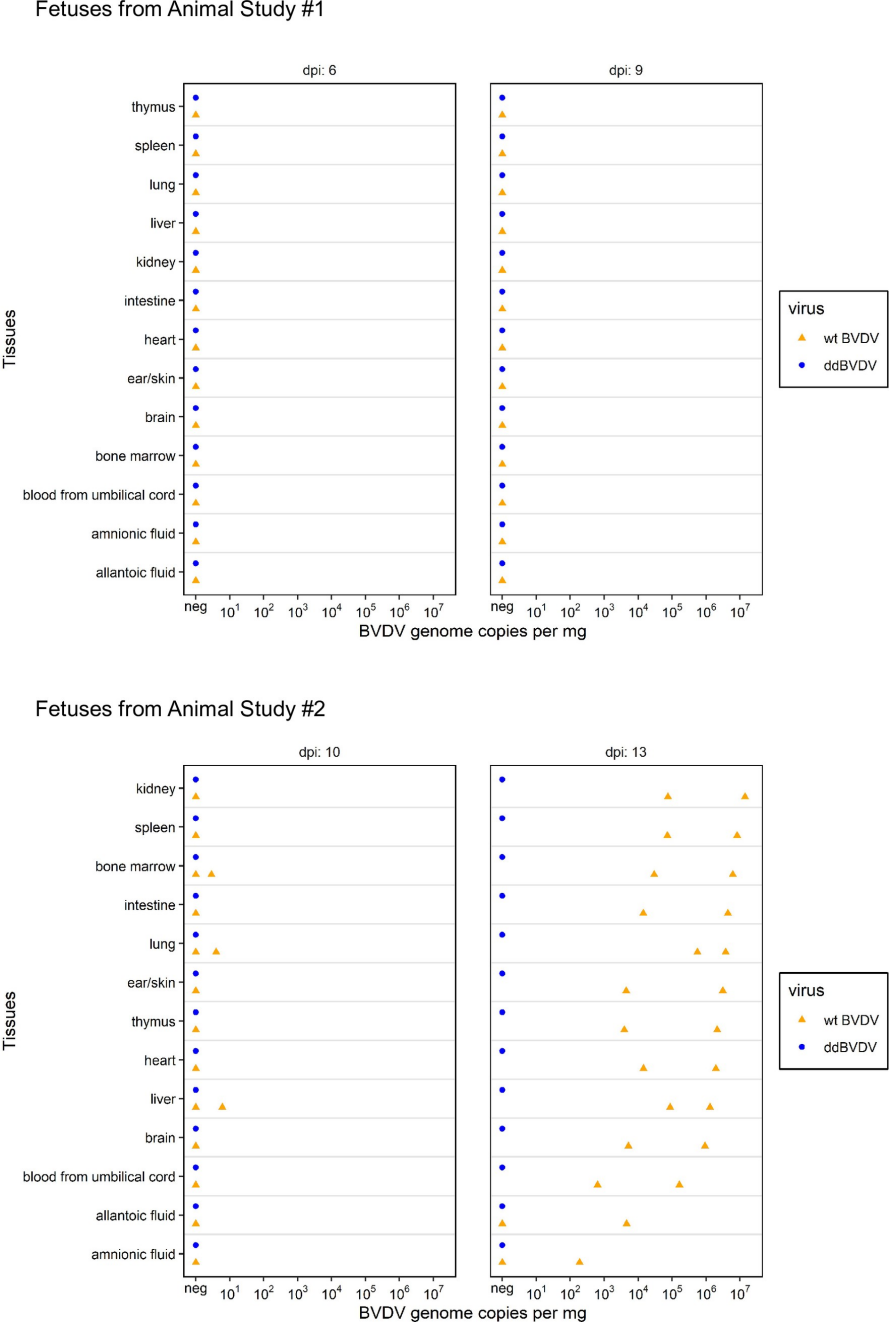
Detection of viral RNA in the tissues taken from fetuses at the indicated time points. Orange triangles and blue dots stand for the animals infected with wt BVDV or ddBVDV, respectively. The positioning of the triangles and dots reflects the amount of viral RNA detected in copy number per mg total RNA.

**Fig 4:**
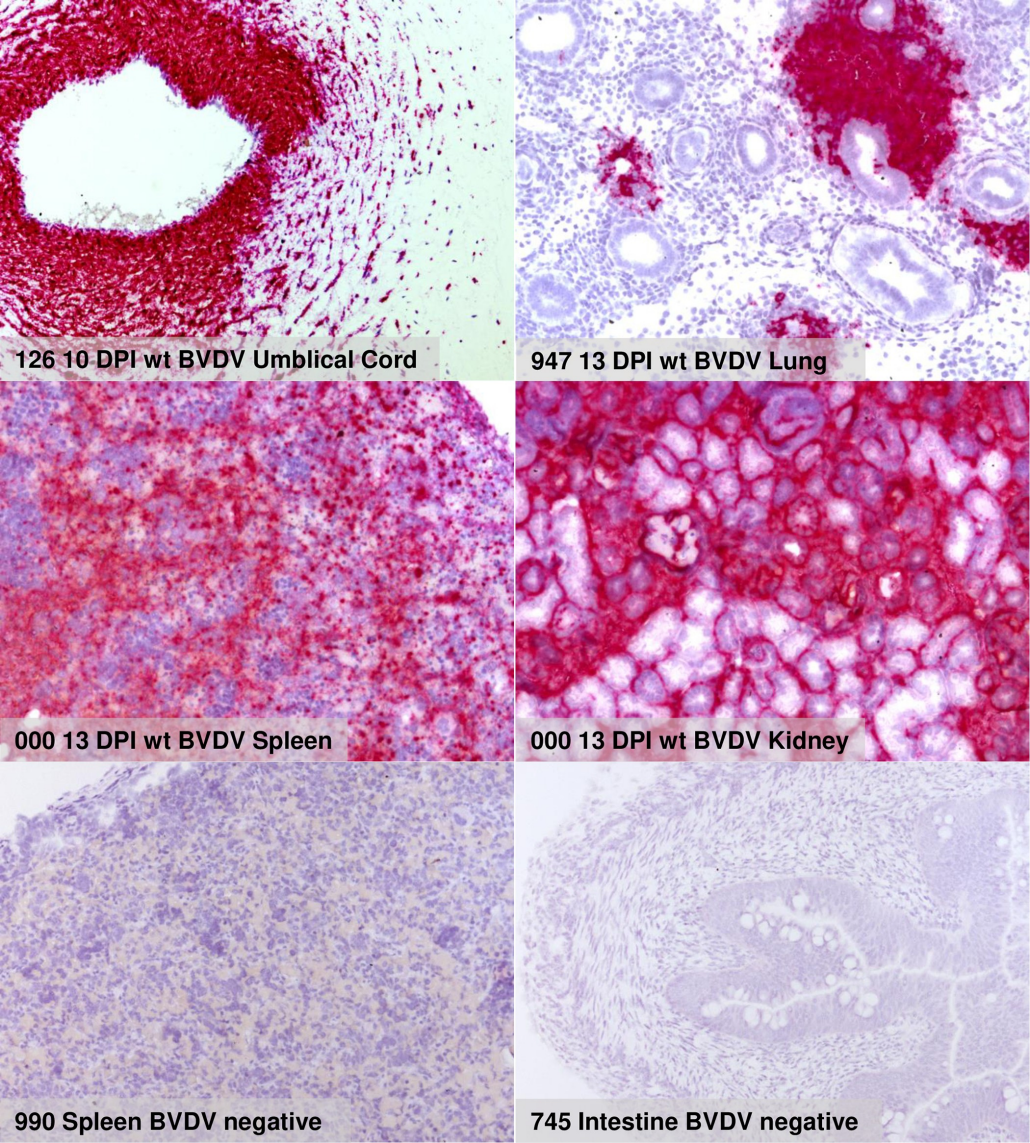
Detection of viral RNA in fetal tissues via RNAscope. The pictures represent the results of RNAscope in situ hybridization of the indicated tissues. The labelling lists from left to right the animal number, the day of slaughter, the virus used for infection and the tissue from which the sample was taken. Red color represents positive staining for BVDV genomic RNA.

Two heifers from the same virus group were slaughtered at each time point (6 and 9 days p.i. in study #1, 10 and 13 days p.i. in study #2). The time points 6 and 9 days p.i. were chosen since in the above mentioned earlier dissemination study in calves ddBVDV antigen was detected only at the early time points. Based on the results of study #1 time points 10 and 13 days p.i. were chosen for study #2. The uteri with the fetuses were extracted immediately and transferred to a clean table for sampling of the placenta (placentomes, caruncles, and cotyledons), amnion, and allantois (Fig 2). The fetus was then removed from the uterus and transferred to a biosafety cabinet in the necropsy hall. The fetus was dissected, and samples of key organs were taken for diagnostic PCR and histopathology (see Figs 3 and 4).

### Detection of virus in fetal tissue

In earlier infection studies with pregnant heifers we already showed that 2 month p.i. our ddBVDV mutant was not found in the fetuses whereas virus was found in 100% of fetuses from heifers infected with wt BVDV (referred to as BVDV KE9 in [53]). In the present study, infection of heifers was done during the same period of highest probability for transplacental transmission but the fetal samples were taken already a few days p.i. RNA was prepared from the different tissues and analyzed with a validated real time RT-PCR protocol [58]. All samples from fetuses in heifers infected with ddBVDV were tested negative. Similarly, also wt virus could not be detected in samples taken on days 6 and 9. However, at day 10 p.i. first weakly positive results showed up and at day 13 all but two tested tissues were positive with viral genome equivalents of up to 10^7^ per mg of tissue which is summarized in a diagram (Fig 3).

Selected formalin fixed issue samples of the fetuses were also tested via RNAscope in situ hybridization for the presence of viral RNA. We did not find inidications of cells positive for ddBVDV RNA but at later time points huge numbers of positive cells were found in fetuses from wt BVDV infected heifers (examples shown in Fig 4). Taken together, the results received for the fetuses here correspond to the above mentioned earlier data obtained 2 months p.i. proving that ddBVDV does not enter the fetus upon infection of a pregnant heifer.

### Detection of virus in maternal tissue

**Fig 5:**
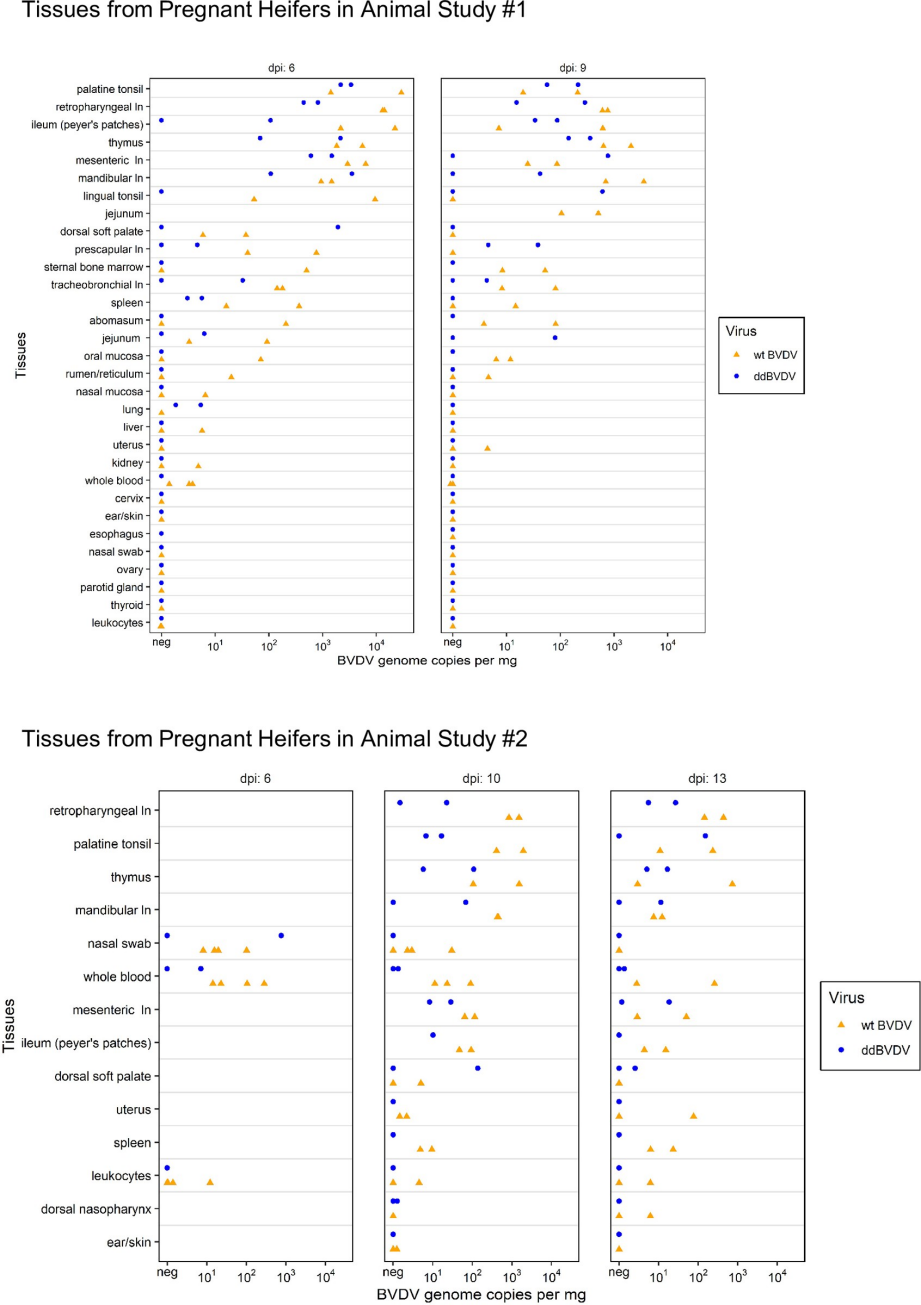
Detection and quantification of BVDV genome in tissues of the heifers. Orange triangles and blue dots indicate the animals infected with wt BVDV or ddBVDV, respectively. The positioning of the triangles and dots reflects the amount of viral RNA detected in copy number per mg total RNA.

**Fig 6:**
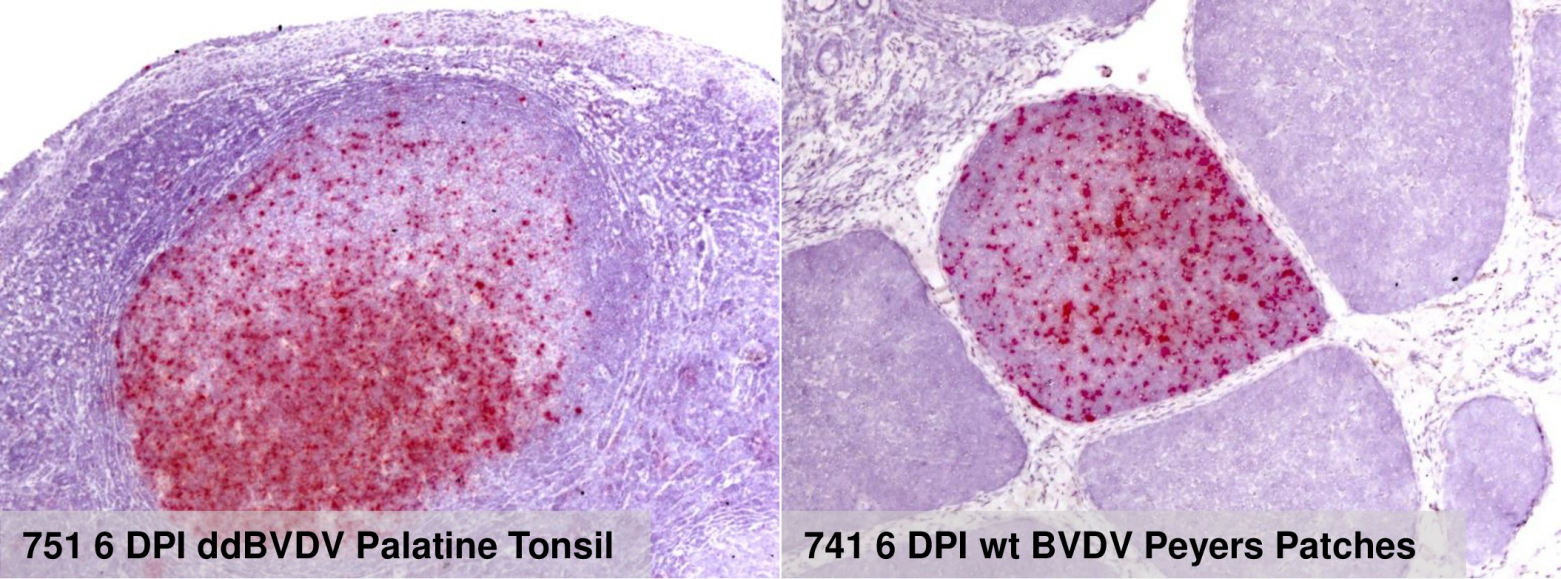
Detection via RNAscope of genomic RNA of ddBVDV in the palatine tonsil of animal 751 and of wt BVDV in Peyers Patches of animal 741, respectively (both from necropsy at 6 DPI). The labeling lists from left to right the animal number, the day of slaughter, the virus used for infection and the tissue from which the sample was taken. Red color represents positive staining.

Also the tissue samples taken at necropsy from the heifers were analyzed for the presence of viral genome via RNA extraction and real time RT-PCR. We analyzed a large variety of tissues from the animals of study #1 (Fig 5, upper part). Since pestiviruses target cells especially from the immune system [59–61],and many samples from lymphoid organs mostly tested positive, we decided to focus the analyses on these organs to reduce the number of tissues collected for study #2. The diagrams summarizing the results show that a lot of tissues from the heifers were infected already at day 6 p.i. (Fig 5). Importantly, this is true not only for wt BVDV but also for ddBVDV. For both viruses the observed genome loads were up to 10^4^ per mg of tissue with high values especially in organs belonging to the immune system. The viral RNA load did not increase from day 6 to later time points p.i., one could even deduce a tendency to reduced values after longer infection which could be due to the beginning of the adaptive immune reaction in the heifers (Fig 5A versus 5B). Equivalent results were also obtained when tissue samples were analyzed via RNAscope: strongly positive results for wt and ddBVDV in tissue samples collected as early as day 6 p.i. (Fig 6). These data also prove that from a technical point of view the RNAscope probe recognizes the ddBVDV genome.

### Analysis of placenta samples

**Fig 7:**
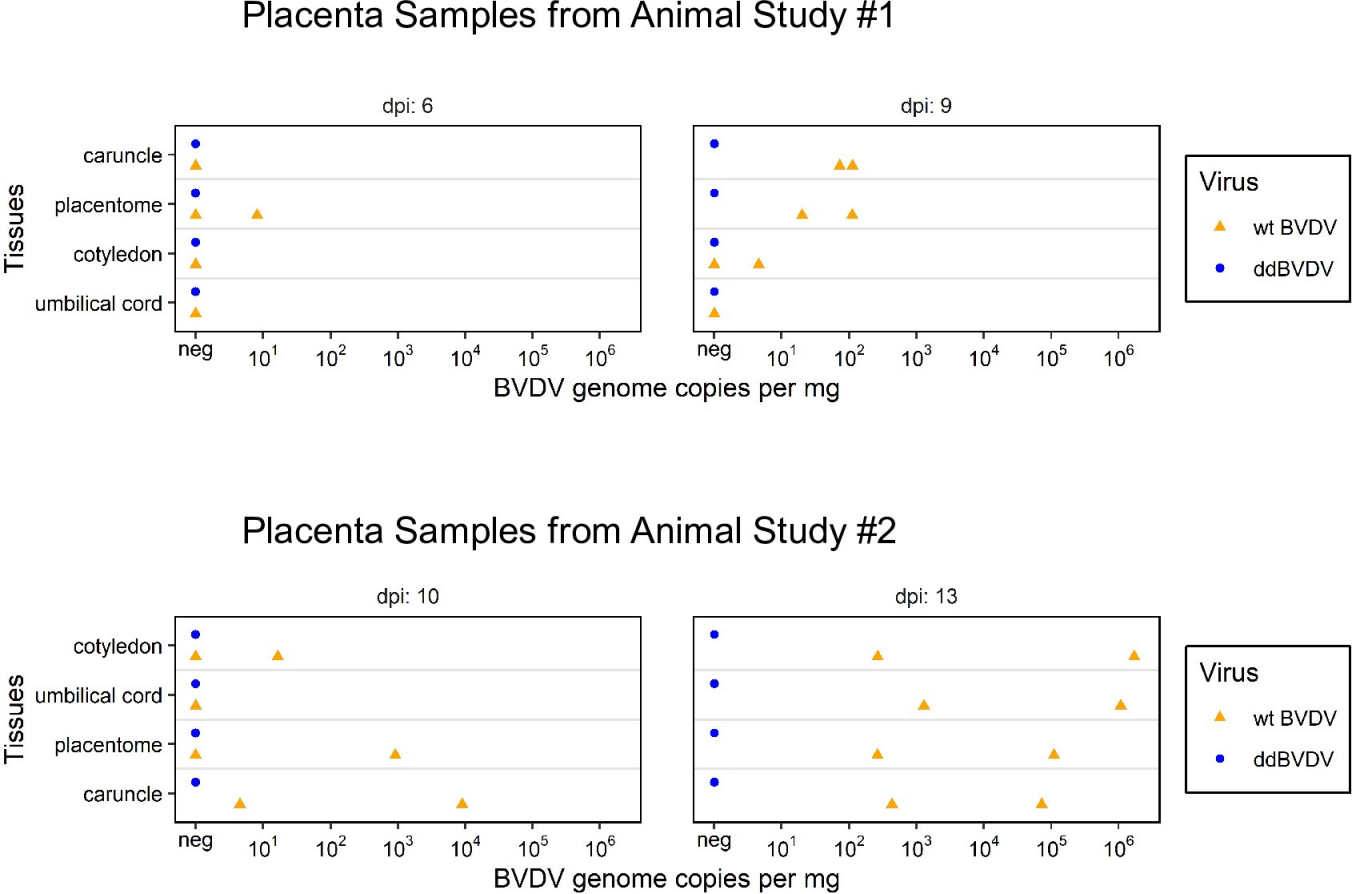
Detection of viral RNA in placenta samples. Orange triangles and blue dots stand for the animals infected with wt BVDV or ddBVDV, respectively. The positioning of the triangles and dots reflects the amount of viral RNA detected (in copies per mg total isolated RNA).

These results support the earlier data showing that ddBVDV is able to replicate and disseminate in the mother animal but does not reach the fetus. The maternal/fetal interphase is located in the placenta within the placentomes where the maternal caruncle is in contact with the fetal cotyledon. During necropsy, samples of whole placentomes were taken but placentomes were also pulled apart to separate the caruncle and cotyledon in order to analyze both sides of the interface independently. We found low but increasing infection rates in the samples derived from animals infected with the wt virus with a tendency towards higher loads of viral RNA in caruncle compared to cotyledon at earlier time points (Fig 7, Table 2). In contrast, the samples derived from the animals infected with ddBVDV were all negative.

**Table 2:** Summary of the results obtained for the placenta samples from the wt virus infected animals via qRT-PCR and *in situ* hybridization.

Animal ID	Tissue	Day of Gest.	dpi	Cq	BVDV PCR	In situ Hybridization
**741**	caruncle	77	**6**	>42	**-**	Undetected
	**cotyledon**			>42	**-**	
	**placentome**			>42	**-**	
**745**	**caruncle**	78	**6**	>42	**-**	Undetected
	**cotyledon**			>42	**-**	
	**placentome**			38.9	**+**	
**685**	**caruncle**	77	**9**	35.1	**+**	**Pos**
	**cotyledon**			>42	**-**	
	**placentome**			37.6	**+**	
**719**	**caruncle**	79	**9**	35.7	**+**	Undetected
	**cotyledon**			39.7	**+**	
	**placentome**			35.1	**+**	
**126**	**caruncle**	80	**10**	38.5	**+**	Undetected
	**cotyledon**			>42	**-**	
	**placentome**			>42	**-**	
**519**	**caruncle**	80	**10**	27.1	**++**	**Pos**
	**cotyledon**			36.6	**+**	
	**placentome**			30.5	**++**	
**000**	**caruncle**	84	**13**	23.9	**++**	**Pos**
	**cotyledon**			19.2	**+++**	
	**placentome**			23.4	**++**	
**947**	**caruncle**	85	**13**	31.6	**++**	**Pos**
	**cotyledon**			32.4	**+**	
	**placentome**			32.4	**+**	

**Fig 8:**
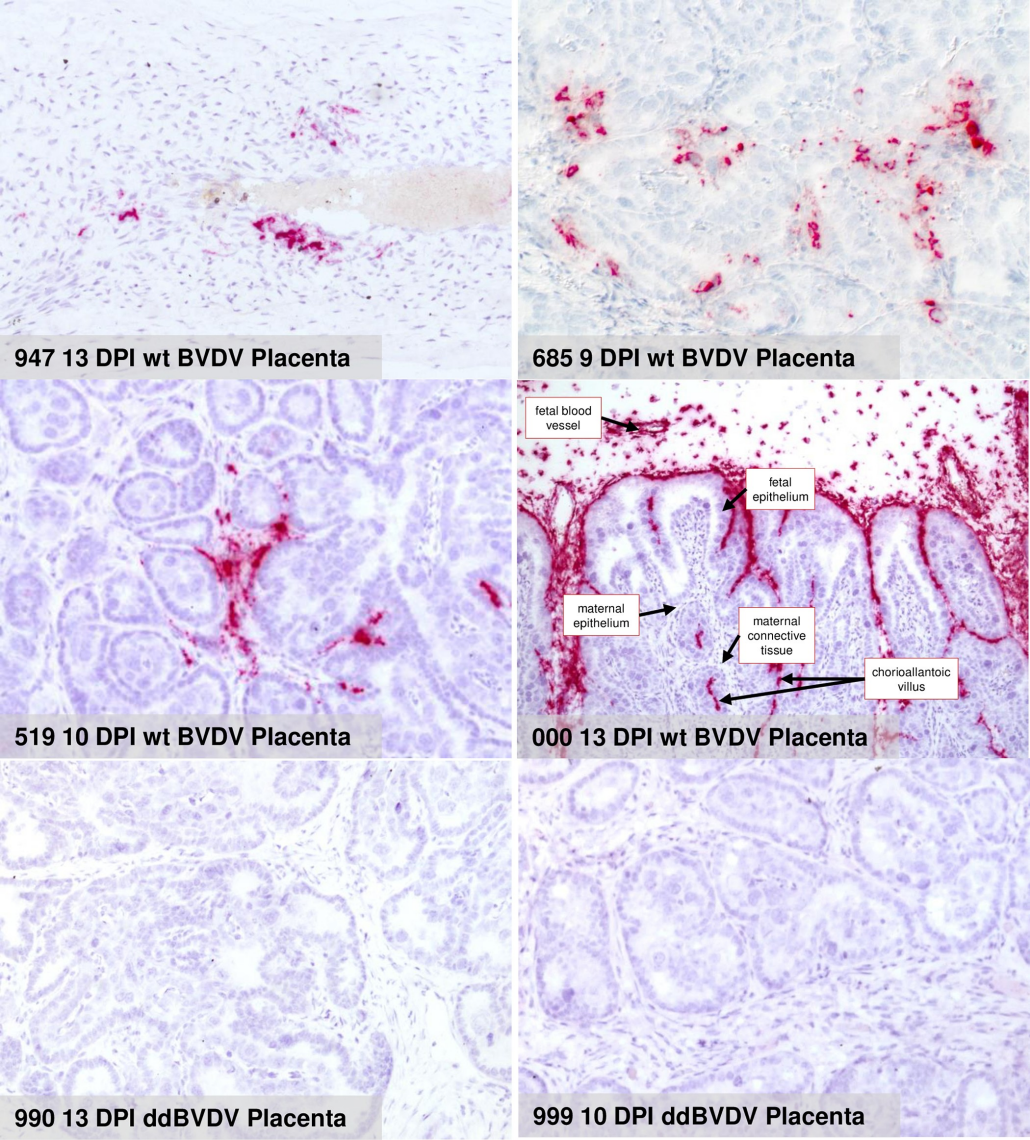
Detection via RNAscope of BVDV genomic RNA in placenta tissue. The pictures represent the results of RNAscope in situ hybridization of the specified placenta samples. The labeling lists from left to right the animal number, the day of slaughter, the virus used for infection and the tissue from which the sample was taken. Red color represents positive staining. In the right picture in the middle row inscriptions and arrows highlight the structural organization of the placenta.

Taken together, the real time RT-PCR analyses already showed that the animal study was successful in terms of inoculation, infection, and viral distribution in the heifers. A significant difference was observed regarding to the viral RNA content between the wt virus and the ddBVDV mutant when samples from the placenta were analyzed with the genome of the mutant virus staying below the detection level. It could, however, not be excluded that single cells or small areas in this organ contained viral RNA which finding could be interesting for elucidation of cell types and/or sites at which the mutant virus replicated but was trapped and prevented from further spread. We therefore used the RNAscope approach also for investigation of placenta tissue at single cell level. Importantly, we were not able to detect the genome of the BVDV double deletion mutant in tissue derived from the placenta of infected heifers or in the fetus. In contrast, the placenta from wt BVDV infected animals gave positive results on days 9, 10 and 13 (Fig 8, Table 2). At least at later time points most of the viral RNA detected via the in situ hybridization is located in the fetal tissue, especially fetal blood vessels, mesenchymal soft tissue cells of the wharton’s jelly, and the basement membrane zone of the fetal epithelium of the cotyledons just at the contact surface with the maternal caruncle tissue (Fig 8, right part, middle section). These findings reflect the results of the real time RT-PCR analyses and point towards a defect of the ddBVDV to replicate in the placenta or a decrease of viral RNA synthesis below the detection limit due to cellular defense mechanisms.

### Array analyses

**Fig 9:**
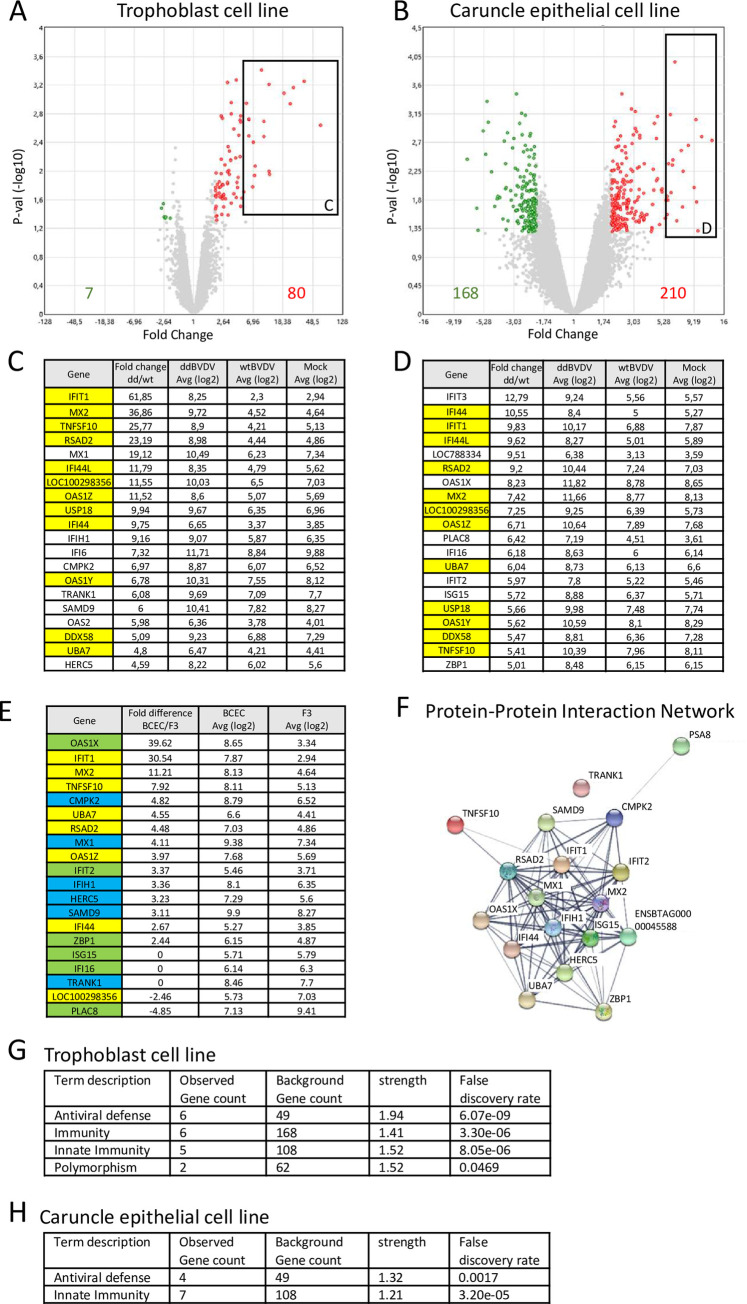
Transcriptional changes in the trophoblast cell line F3 and the caruncle epithelial cell line BCEC-1 upon infection with wt BVDV and ddBVDV. (A) Vulcano plot showing the statistical significance of differentialy regulated genes of the trophoblast cell line F3 infected either with ddBVDV or wt BVDV. Genes in the rectangle are shown in C. The number of genes downregulated or upregulated by more than twofold are given in the graph (left and right bottom, respectively) (B) Vulcano plot showing the statistical significance of differentialy regulated genes of the caruncle epithelial cell line BCEC-1 infected either with ddBVDV or wt BVDV. Genes in the rectangle are shown in E. Meaning of numbers given in the graph are same as in (A) (C) The relative expression of the 20 genes with the highest “fold change” in F3 cells are shown. (D) The relative expression of the 20 genes with the highest “fold change” in BCEC-1 cells are shown. Genes showing up as differentially expressed in both F3 and BCEC-1 cells are highlighted in yellow (E) Comparison of gene expression levels in non-infected BCEC-1 and F3 cells. Expression levels for the two cell lines are given as log 2 in columns 3 and 4, respectively, whereas column 2 shows the ratio of the levels found in BCEC-1 and F3. Genes found among the 20 highest regulated genes in both cell lines are highlighted in yellow, whereas blue highlights genes found only in F3 cells (C) and green labels those found only in BCEC-1 (D). (F) The interactome of the genes listed in (E) (G, H) Gene enrichment analyses of upregulated genes (≥ 3 fold) of F3 (G) and BCEC-1 (H) using DAVID software.

The experiments described above revealed the absence of ddBVDV RNA from the placenta of the pregnant heifers whereas wt BVDV propagation was demonstrated. Since the virus mutant was able to replicate in other tissues of the mother animals as shown by both real time RT-PCR and RNAscope analysis (Figs 5 and 6), the results pointed at a placenta specific infection or replication defect of the mutant thereby preventing accumulation of detectable levels of viral RNA. Both deletions introduced into ddBVDV affect proteins engaged in blocking the innate immune response to virus infection. The placenta is known for its high level of innate immune functions so that it was worthwhile to compare the responses of placenta cells to infection with wt BVDV and ddBVDV. Since we were not able to detect the virus mutant in placenta tissue of the animals in our infection study a comparison of gene expression in placenta tissue of wt BVDV versus ddBVDV infected animals would *de facto* result in comparison of wt BVDV infected with noninfected tissue and thus give misleading impressions. We, therefore, decided to use for these studies well characterized cell lines established from a bovine placenta, namely caruncular epithelium cell line BCEC-1 and trophoblast cell line F3 [62–64] (see also Fig 2 for illustration of the origin of the cells). While the caruncular epithelial cells represent the maternal side of the maternal/fetal border, the trophoblast cells are located on the fetal side (see also Fig 2). We infected both cell lines with either wt BVDV or ddBVDV at a MOI of 1. Immunofluorescence analysis conducted 48h post infection revealed that nearly 100% of the cells got infected and expressed viral proteins. To get an impression of the host cellular reaction to virus infection we isolated RNA at 48 h p.i. from the infected cells, and conducted a microarray analysis. This time point was chosen since it was appropriate for our main focus, namely the expression of interferon and interferon stimulated genes because both N^pro^ and E^rns^ RNase are known to interfere at this key reaction of innate immunity as already mentioned above. Moreover, the cells show nearly complete infection at this time point, which is helpful for the analysis. For both cell lines comparison of the results obtained for noninfected controls and wt virus infected cells did not reveal strong infection dependent effects. Importantly, the low number of poorly induced genes did not indicate an antiviral response signature demonstrating this result is in agreement with the hypothesis that N^pro^ and the E^rns^ RNase block such a response. However, significant differences between cells infected with the mutant versus cells infected with the wt virus were observed for both cell lines. The ddBVDV mutant induced much higher levels of expression of a large variety of genes (Fig 9A and 9B) among which a high number of transcripts coding for components of the innate immune system like IFIT1, IFIT3, ISG15, MX1, USP18 and OAS1Z (Fig 9C–9H) was detected. Accordingly, gene enrichment analyses identified the highest enrichments of genes belonging to the term “antiviral defense”, “immunity” and “innate immunity”. Thus, the presence of N^pro^ and E^rns^ RNase in wt BVDV obviously was able to control most of the innate response to infection whereas the deletions affecting these viral factors in the ddBVDV mutant eliminated this repression process. As a further general result we observed a higher reactivity of the BCEC-1 cells in comparison to F3. The induction of gene expression as well as the down-regulation of genes affected a higher number of genes in BCEC-1. Furthermore, BCEC-1 cells showed a higher expression of innate response genes including IFIT1, IFIT3, MX2, OAS1Z and RSAD2 than F3 cells already without virus infection (Fig 9E) and in particular upon wt BVDV infection (Fig 9C and 9D columns 5 and 4, respectively, and Fig 9E). Nevertheless, infection with the ddBVDV pushed the expression of these genes in BCEC-1 cells to even higher levels when absolute levels were regarded. In comparison, F3 cells showed enhanced expression of a lower number of genes but with higher maximum fold changes (Fig 9A and 9C). Since wt infected cells showed similar expression rates of the antiviral response genes as noninfected cells these findings indicate that BCEC-1 cells have a higher steady state level of expression of genes belonging to the innate immune system.

**Fig 10:**
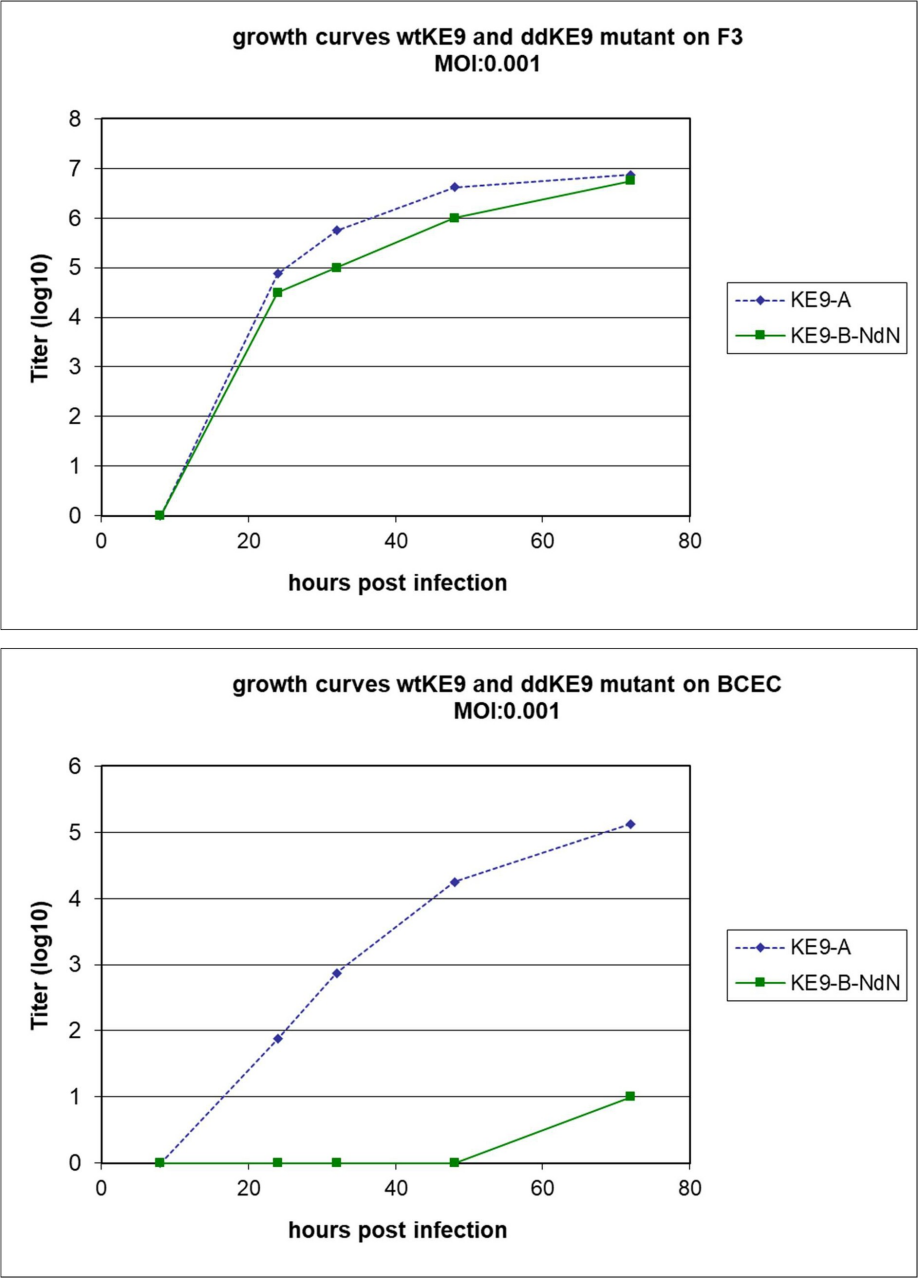
Growth curves of wt BVDV and ddBVDV mutant on bovine placenta cell lines. Cells of the lines indicated on top of the diagrams were infected with a MOI of 0.001 with either of the two viruses. Freeze/thaw extracts of infected cultures were prepared at the indicated time points p.i. and viral titers were determined as described [53,79] with detection of infected cells via indirect immunofluorescence using monoclonal antibody code 4 (mAb 8.12.7 [87]) and FITC anti mouse as secondary antibody.

The results described above were in agreement with the expected different features of our viruses concerning their effects on the innate immune system but could not explain why both viruses replicated to detectable amounts in the cultured placental cells whereas in the animal ddBVDV was found in all tested maternal tissues but not in the placenta, in which only the wt virus was found. We speculated that in cell culture experiments the high amount of virus used in these experiments did not appropriately mimic the natural situation in the animal where only low numbers of viruses can be expected to enter the placenta. Therefore, another infection experiment was conducted with infection at a very low MOI of 0.001 and subsequent analysis of the growth characteristics of both viruses on both cell lines (Fig 10). Surprisingly, we observed significant differences between the two cell lines. In the F3 trophoblast cells both viruses replicated at rather similar rates with a slight retardation for the ddBVDV mutant. Both viruses reached equivalently high titers at 72 h p.i. In contrast, growth of both viruses was significantly hampered in the BCEC-1 caruncular epithelium cells compared to the F3 results. For the wt BVDV the titer was at least two logs lower at any time point compared with the F3 cell results and did obviously not reach a steady state within 72 h. This observation could be explained with the higher steady state expression levels of innate immune factors in these cells compared to F3. Even more importantly, the replication of the ddBVDV mutant was inhibited almost completely. The only positive result was obtained after 72 h with a log TCID_50_ of 1 per ml. Thus, the caruncular epithelial cells significantly interfered with BVDV replication in general and loss of the N^pro^ and E^rns^ RNase dramatically enhanced this effect.

## Discussion

The strategy underlying the establishment of lifelong persistent infections with BVDV represents one of the most fascinating stories in virology and demonstrates the delicate adaptation of virus and host system developed during evolution [5]. The main step leading to persistent infection is the transfer of virus in pregnant heifers via the placenta to the fetus. Because of the structure of the bovine placenta, virus replicating in the fetus is not accessible to maternal immune control [65]. When the virus enters the fetus during the first trimester of gestation it starts replication in a host that is not immunocompetent. Then later on in embryonic development the immune system maturates and learns to discriminate self from non-self antigen the virus is already there and is, therefore, regarded as self so that the host system is not mounting an adaptive immune response to the virus [66–68]. Replication of the persisting virus in the fetus impairs fetal development to some extent and has also an effect on the maternal immune system [69]. Due to its design as a pattern recognizing system, the innate immune response to virus infection cannot be silenced by early infection of the fetus. Thus, infection at an appropriate timepoint of pregnancy is a prerequisite for establishment of persistence but control of the innate system is also crucial. Evolution has provided BVDV with three factors that help to reduce the innate immune response in the infected fetus to an acceptable level. As a first point, BVDV strictly controls its RNA replication so that the double stranded RNA serving as a trigger for an interferon response via e.g. RIG-like pattern recognition receptors is only present in the infected cell at low levels [5,70–72]. This restriction of RNA replication is only observed for non-cytopathic BVDV and is due to the fact, that generation of the essential replicase component NS3 is dependent on the low abundant host factor JIV that is exhausted while NS3 is generated. Second and third are two factors, N^pro^ and E^rns^ RNase, that block the induction of an interferon response in experimental systems [5,53,73]. Interestingly, wt BVDV can, nevertheless, induce a significant interferon response in the infected adult host, but no detectable or very low responses in the persistently infected fetus [68,69,74,75]. Elimination of N^pro^ and E^rns^ RNase activity resulted in ddBVDV that replicated in the mother animals and induced a virus specific immune response but was not found in the fetus of such heifers [53]. Obviously, ddBVDV was not able to establish persistent infection in fetuses of infected heifers. In contrast, circumvention of the maternal system via introduction of BVD virus to the amniotic fluid resulted in death of the fetuses and detection of virus in fetal tissue proving that ddBVDV can replicate in the fetus [53]. These findings led to the conclusion that ddBVDV is not able to cross the placenta upon infection of pregnant heifers. Earlier studies analyzing virus dissemination in calves (not shown) and the present experiment in pregnant heifers prove that ddBVDV not only replicates locally but spreads to a variety of different organs of the heifer. The early dissemination kinetics and the virus yields determined for the various tissue samples were rather similar for wt and ddBVDV. Thus, the defect of ddBVDV transmission to the fetus was hypothesized to be due to a specific barrier hindering transplacental spread. This hypothesis is proven by our present work since not only the fetus but also the placenta was free of ddBVDV RNA indicating that replication of the virus mutant was blocked in the placenta tissue.

Pregnancy represents an immunological threat (see [76,77] for recent reviews). Fetal antigens are allogeneic to the maternal immune system and should, therefore, induce an immune response resulting in rejection by the mother’s immune system. However, this does not occur even though the fetal antigens are present over a long time, and the reason for this fact is still a scientific mystery. One important prerequisite helping to prevent rejection of the fetus is a partial repression of the maternal immune system during pregnancy. However, this partially immunocompromised state must not significantly impair the ability of the maternal immune system to control invading pathogens to protect mother and the immunologically incompetent fetus. The bovine placenta belongs to the epitheliochorial type which is characterized by a strict separation of maternal and fetal system and excludes the transfer of antibodies [65]. Thus, a pathogen cannot be eliminated from the early fetus once having crossed the maternal/fetal interface, so that prevention of transmission has to be insured to protect the fetus. When pregnancy induced partial dampening of the adaptive immune system is necessary the innate system must fill the gap and trap pathogens before transmission to the fetus. Accordingly, the innate immune system has to be highly active in the placenta, however, BVDV of the noncp biotype controls its RNA replication and furthermore expresses N^pro^ and E^rns^ RNase as efficient repressors of the interferon response, so that it can circumvent the placental barrier and reach the fetus. We demonstrate here, that elimination of the viral factors repressing the interferon response prevents viral replication in the placenta and thus blocks the transfer to the fetus.

Our data clearly show that virus detection and dissemination in fetal samples diminishes considerably compared to the viral dissemination in the mother animals. In the maternal tissues considerable amounts of viral RNA were detected already on day 6, regardless of the virus used for infection. Since viremia becomes detectable in BVDV infected animals around day 3 p.i. [78–80] the infection of the tissues can be regarded as a result of virus dissemination from the primary site of infection. In the placenta of the wt BVDV infected animals the first very weakly positive sample was also from day 6 p.i. Starting from day 9 p.i. placenta samples were clearly positive but still only for the wt virus. The apparently lower or delayed infection of the placenta compared to other organs might reflect a retarded infiltration by the virus or, more likely, a stronger repression of viral replication in this organ. Importantly, the first detection of low amounts of viral RNA in three fetal samples from the wt BVDV infected heifers was at day 10 p.i. At day 13 p.i. all fetuses from wt infected heifers contained high amounts of viral RNA, which are about 3 logs higher than all values determined for any maternal tissue. It can be hypothesized that transfer via the placenta is delayed (maybe because of restricted viral replication in the placenta due to the high level of innate immunity in this tissue) and/or that only an extremely limited number of virus particles cross the placental barrier so that several days of replication are needed to generate detectable amounts of viral RNA. But then, the virus titers in the wt BVDV infected fetuses dramatically increase, because of the strongly compromised immunological control. It seems likely that the increase in wt virus genome concentration in the placenta at later time points (especially day 13 p.i.) is due to transfer of virus from the fetus back to the dam, which is also suggested by the intense RNAscope staining at the maternal/fetal interphase (Fig 8) at a time point, at which most fetal tissues react strongly positive in real time RT-PCR.

The reason for the obvious repression of ddBVDV replication in the placenta of pregnant heifers is still obscure. Our data clearly show that the double deletion mutant can replicate in other maternal tissues despite the absence of the two known factors engaged in repression of the type 1 and type 3 interferon response. Similarly, strong replication of ddBVDV in fetal tissues upon direct infection of the fetus via the amniotic fluid was demonstrated [53]. There is no logical reason to believe that the interferon induced innate immune system is not active in the cells of the positively tested tissues and at least for the directly infected fetuses a very strong IFN response was demonstrated which is most likely the reason for the observed death of such fetuses [53]. The activation of an innate immune response was also observed in the array analyses of RNA from ddBVDV infected BCEC-1 cells as some of the key players of the interferon system were upregulated. However, also in non-infected and wt virus infected BCEC-1 cells increased expression levels of these genes compared to the F3 cells were detected indicating a higher reactivity of the caruncular cells. One must keep in mind that in the initial phase of a natural infection only very low amounts of virus reach a specific organ, so that an increased innate immune response to the double deletion mutant could be responsible for repressed replication of the virus in the placenta, whereas the response to viral infection is too low to control virus replication in other tissues. This placenta specific response would explain the retarded detection also of wt BVDV RNA, and, indeed, the growth curve shows a clearly reduced replication rate of wt BVDV in these cells. However, this strong antiviral response in the caruncular epithelium can obviously be controlled by the concerted action of N^pro^ and E^rns^ RNase to a level that allows the wt virus to replicate to titers sufficient for crossing the maternal/fetal barrier. This hypothesis is supported by the gene array analyses showing that compared to noninfected cells infection of the placenta-derived cell lines with wt BVDV does not induce strong changes in gene expression, and, most importantly, these changes do not cluster in antiviral response genes. In contrast, the ddBVDV mutant induces high expression of antiviral genes which in the BCEC-1cells adds to a high steady state expression level and, therefore, blocks replication of the mutant almost completely. We show here for the first time that the viral factors N^pro^ and E^rns^ are not only necessary for establishment of persistent fetal infection in the fetus but also crucial for BVDV replication in the placenta, which is a prerequisite for the transfer of virus from the mother to the fetus. This finding adds a new item to the complex and fascinating story of a virus that learned its lessons extremely well.

## Material and methods

### Ethics statement

The animal experiments were approved by the State Office of Agriculture, Food Safety and Fishery in Mecklenburg-Western Pomerania (LALFF M-V) with reference number LALLF7221.3-1-072/17 and discussed with members of the IACUC.

### Cells and viruses

BVDV KE9-A (named wt BVDV throughout the text) and BVDV KE9-B-NdN (double deletion mutant–named ddBVDV in the present manuscript) were both recovered from infectious full length cDNA constructs via *in vitro* transcription and RNA transfection [53]. The parental BVDV KE9 strain was isolated during a routine screening of German BVDV field strains. It was selected for further work because it is efficiently transmitted to the fetuses in pregnant animals. The viruses were propagated in MDBK-B2 cells, a cell clone established by end point dilution from MDBK cells (obtained from the American Type Culture Collection (Rockville, Md.)) grown in Dulbecco’s modified Eagle’s medium supplemented with 10% fetal calf serum (FCS; tested for the absence of pestivirus and antibodies against pestiviruses) and nonessential amino acids. The B2 clone was selected for further work because of its superior properties in transfection experiments. MDBK-B2 cells were shown to mount a type I IFN response after e.g. infection with cytopathogenic BVDV (not shown).

BVDV infection of tissue culture cells was detected via indirect immunofluorescence assay using monoclonal antibody code 4 (mAb 8.12.7 [81]) and FITC anti mouse as secondary antibody as described before [53].

Bovine placental cell lines BCEC-1 (caruncular epithelium) and F3 (trophoblast) were isolated from two BVDV-free pregnant cattle with an estimated gestational age of 4 and 5 months, respectively, as described before and grown as reported [62,63,82,83].

### Animal study

The pregnant heifers were purchased from a BVDV-negative farm. Prior to inoculation, proof of pregnancy was obtained by ELISA analysis (Bovine Pregnancy Test Kit ELISA, IDEXX, Kornwestheim, Germany), and the absence of BVDV and anti BVDV antibodies was certified by the BVDV reference lab at the FLI (Kerstin Wernike, Institut für Virusdiagnostik, Friedrich-Loeffler-Institut, Insel Riems). After acclimatization the heifers were inoculated intramuscularly with 10^6.0^ TCID_50_ in 1 mL cell culture medium with wt BVDV or the ddBVDV mutant at 66–72 days of gestation. The heifers were examined daily throughout the trial. The lack of clinical signs was recorded for the group. Change of behaviour, appetite, and the presence of clinical signs were recorded for individual animals using a clinical score. The score system was based on five parameters reflecting the known possible clinical signs after BVDV infection (general health, behaviour, respiration, cough, rectal temperature). Based on experience with the viruses intended for the trial, it was assumed that no animal would reach a total score of 5 or more in the study. Rectal temperatures were measured daily in each heifer from 7 days prior to the inoculation till 8 days post-inoculation (p.i.). Blood was collected in heparin, EDTA and serum tubes at days 0, 6, and 9 or 0, 6, 10, 13 p.i. (first or second study, respectively). Two heifers from each group were humanely slaughtered at days 6 and 9 or 10 and 13 post-infection (first or second study, respectively). After stunning by captive bolt, the heifers were fully exsanguinated. The fetuses within the uterus were carefully extracted to avoid contamination until dissected in a biosafety cabinet to avoid crosscontamination. The placenta and other organs were collected from the heifers for virus genome detection via qRT-PCR, RNAscope *in situ*-hybridization, and RNA isolation for gene expression studies by gene array analysis.

### Preparation of Buffy Coats

Heparin blood samples were taken and transported to the laboratory as soon as possible. Blood was spun down, and plasma was removed. Ten milliliters cold lysis buffer (154 mM NH_4_Cl, 7.3 mM KHCO_3_, and 1.3 mM EDTA) was added to red cells and incubated for 10 mins on ice. The lysed cells were then centrifuged for 5 min at 1800 rpm at 4°C. Leukocytes/ buffy coat were washed twice in Phosphate Buffer Saline (PBS) and then resuspended in PBS with 10^7^ cells per mL. The buffy-coat samples were transferred to 2 cryovials and frozen at –70°C.

### Nasal swabs

Nasal swabs were collected with a tampon. The tampon was inserted into the nasal passage to soak up any nasal secretions. One mL of PBS was added to the tampon and they were spun down at 3,000 rpm for 10 minutes at 4°C. All samples were PCR negative for BVDV genome.

### Tissue sample preparation for viral RNA detection via real time RT-PCR

Portions of organ tissue (less than 250 mg) were placed in a 2 mL Eppendorf tube with a sterile steel ball and 500 μL of DMEM medium with glutamine and 1% antibiotics were added. Tissues were homogenized in a Qiagen TissueLyser for 2 minutes at 30 Hz. The homogenized sample sat on ice for 1 hour and then was centrifuged at 1215 x g for 15 min at 4°C. From the supernatant, 100 μL was used for the Macherey-Nagel King Fisher 96 Flex extraction (Düren, Germany) processed on a King Fisher extraction roboter (ThermoFisher Scientific, München Germany) according to manufacturer’s protocol. 100 μl of EDTA blood or tissue homogenate was used for extraction. After extraction, RNA was eluted in 100 μl and 5 μL were used for the “panpesti” real-time RT-PCR [58] with beta-actin RNA detection serving as a control [84]. The undiluted homogenates were stored at -70°C for future virus isolation.

Subsequently, nucleic acids were analyzed using a published real-time RT-PCR assay targeting the 5’ NTR region described in Hoffman et al 2006 [58], in combination with an internal control based on beta actin described by Toussiant et al. 2007 [84] on a CFX96 real-time cycler (Bio-Rad Laboratories, München, Germany) (primer sequences given in Table 3). PCR was performed with the SuperScrip III One-Step RT-PCR System with Platinum Taq DNA Polymerase (Invitrogen) in a total volume of 20 μL, with 10 pmol of each BVDV primer, 1.25 pmol of the BVDV probe, 2.5 pmol of each beta actin primer and 2.1 pmol of the beta actin probe. The temperature profile was 30 min reverse transcription 50°C, 2 min initial activation at 94°C, followed by 42 cycles of denaturation for 30 sec at 94°C and annealing 60 s at 57°C and elongation 60 s at 68°C.

**Table 3:** Real-time quantitative PCR primers.

Primer Name	Sequence 5–3’	Genomic Target
**BVD190-F**	GRA GTC GTC ART GGT TCG AC	**5‘ NTR**
**V326-R**	TCA ACT CCA TGT GCC ATG TAC	
**TQ-Pesti-FAM**	6-FAM-TGC YAY GTG GAC GAG GGC ATG C-BHQ-1	
**ACT2-1005-F**	CAG CAC AAT GAA GAT CAA GAT CAT C	**Beta Actin**
**ACT-1135-R**	CGG ACT CAT CGT ACT CCT GCT T	
**ACT-1081-HEX**	Hex- TCG CTG TCC ACC TTC CAG CAG ATG T -BHQ-1	

Using a dilution series of an BVDV in-vitro transcribed RNA standard, the genome copies in the respective samples were determined to allow harmonization between PCR runs. A standard for BVDV was used to calculate number of genome copies. The RNA concentration was determined by spectrophotometry using a Nanodrop 2000c (ThermoFisher Scientific), and the corresponding number of genome copies was calculated based on the BVDV segment length using an online tool [http://www.molbiol.ru/eng/scripts/01_07.html].

The stock solutions of the *in vitro*-transcribed RNA were stored at working dilutions of 2×10^5^ copies/μl at −20°C in RNA Safe buffer (Carrier-poly A 10 μg/μL, Tween-20 5%, Sodium Azide 5%).

### RNAscope *in-situ* hybridization assay set up

To detect BVDV RNA, RNA in situ hybridization (ISH) was performed on tissues using the RNAScope 2–5 HD Reagent Kit-Red (ACD, Advanced Cell Diagnostics, Newark, CA) according to the manufacturer’s instructions. The probe design was conducted by ACD for wt BVDV. A positive control probe peptidylprolyl isomerase B (cyclophilin B, ppib) and a negative control probe dihydrodipicolinate reductase (DapB) were used to verify specificity of the wt BVDV probe.

As a first step we tested the new probes using paraffin embedded cell pellets established with tissue culture cells (MDBK-B2). The probe reacted well with the RNA of the wt virus and double deletion mutant present in cells that had been formalin fixed and embedded in paraffin whereas no staining occurred with mock infected control cells (not shown). Briefly, tissue samples taken from the animals were embedded in paraffin wax and cut at 5μm sections. Mounted sections were baked on a heating plate (1 hour, 60°C). After deparaffinization, FFPE tissue sections were pretreated with hydrogen peroxide (ACD) (10 min, RT), washed in distilled water and incubated with Target retrieval solution (ACD). Slides were subsequently washed in distilled water and 100% ethanol. After creating a hydrophobe barrier around the tissue section, slides were treated with Protease Plus solution (ACD), incubated in a HybEZ oven (40°C, 30min) and finally washed in distilled water. Next, different hybridization steps were performed using the HybEZ oven according to the protocol provided by ACD. Positive signals were detected after treatment with RED solution (ACD). Slides were counterstained with hematoxylin.

### Array analysis of gene expression

RNA from BCEC-1 and F3 cells infected with wt BVDV and ddBVDV was extracted and purified with a method based on the work of Chrigwin and colleagues [85] as described previously [86]. RNA from 2 independent experiments was used. The quality of the RNA was determined using an agilent bioanalyser. Labeled fragmented single-stranded cDNAs (ss-cDNA) were synthesized by using purified total RNA (100–500 ng) as template following Affymetrix WT PLUS Affymetrix WT PLUS Labeling Assay protocols. Bovine Gene 1.0 ST Arrays (Affymetrix, Santa Clara, CA, USA) were hybridized to the biotinylated ss-cDNA targets. After 20 hours of hybridization at 48°C, arrays were washed by a fluidics station and then scanned by an imaging station in a GeneAtlas System (Affymetrix, Santa Clara, CA, USA). After scanning, the intensity data (CEL files) of Bovine Gene 1.0 ST arrays (Affymetrix) were extracted from the image data (DAT files) by the Affymetrix Command Console Software Version 1.4, and then normalized and analyzed by the Affymetrix Transcriptome Analysis Console (TAC) Software 4.0 for gene expression profiles and differentially expressed genes (DEGs). The DEGs were selected by a cutoff of fold change >2. For gene enrichment analysis we used the Database for Annotation, Visualization and Integrated Discovery (DAVID) https://david.ncifcrf.gov/ and for the analyses of protein-protein interactions the “Search Tool for Retrieval of Interacting Genes/Proteins” STRING v11 https://string-db.org/.
